# Einfluss des Schulrucksackgewichtes bei Grundschulkindern: Gang, Muskelaktivität, Haltung und Stabilität

**DOI:** 10.1007/s00132-020-04047-8

**Published:** 2020-12-09

**Authors:** Anna K. Hell, Lena Braunschweig, Birgit Grages, Reinald Brunner, Jacqueline Romkes

**Affiliations:** 1 Kinderorthopädie, Operatives Kinderzentrum, Klinik für Unfallchirurgie, Orthopädie und Plastische Chirurgie, Universitätsmedizin Göttingen, Robert-Koch-Straße 40, 37075 Göttingen, Deutschland; 2 Universitäts-Kinderspital beider Basel (UKBB), Spitalstrasse 33, 4056 Basel, Schweiz

**Keywords:** Kindergesundheit, Ganganalyse, Schulranzen, Grundschule, Gehgeschwindigkeit, Childrens health, Gait analysis, School back pack, Primary schools, Walking speed

## Abstract

**Hintergrund:**

Das tägliche Tragen eines schweren Schulrucksacks kann möglicherweise Haltungsstörungen hervorrufen, die sich auf das Gangbild und die Standstabilität der Kinder und Jugendlichen auswirken.

**Ziel der Arbeit (Fragestellung):**

Ziel der vorliegenden Studie war es, den Einfluss einer Rucksacklast von 4 kg auf das Gangbild und die Standstabilität bei Grundschulkindern zu analysieren.

**Material und Methoden:**

In der prospektiven Arbeit wurde ein Kollektiv von zwölf Grundschulkindern zwischen 7 und 10 Jahren ohne neurologische oder orthopädische Probleme untersucht. Die Messungen beinhalteten eine klinische Untersuchung, eine dreidimensionale Ganganalyse mit elektromyografischen Aufzeichnungen und die Prüfung des ruhigen Stehens auf einer Kraftmessplatte.

**Ergebnisse:**

Die Rucksacklast von durchschnittlich 15 % des Körpergewichts führte zu reduzierter Ganggeschwindigkeit, verkürzter Schrittlänge und verlängerter Doppelunterstützungsphase. Zudem kam es zu einer erhöhten Oberkörpervorneigung, Beckenkippung und Hüftbeugung. Auch die Muskelaktivität und Stabilität der Kinder wurde durch die erhöhte Traglast beeinflusst.

**Diskussion:**

Schulrucksäcke mit einem Gewicht von 4 kg führten bei Grundschulkindern zu Änderungen von Gang, Muskelaktivität, Haltung und Standstabilität. Das Gewicht des Rucksacks verlagert den Körperschwerpunkt nach hinten und führt zur Instabilität. Dies wird beim Gehen durch eine vermehrte Oberkörpervorneigung, Beckenkippung nach vorne und vermehrte Hüftbeugung kompensiert. Die verminderte Paraspinalmuskelaktivität deutet darauf hin, dass der Rucksack passiv getragen wird. Dies könnte sich im Langzeitverlauf negativ auswirken.

## Abstract (en)

**Background:**

Wearing a school backpack every day may cause postural problems and affect the gait pattern of children and adolescents. The aim of the present study was to analyze the influence of a 4 kg backpack load on the gait pattern and postural sway.

**Objectives:**

The aim of the present study was to analyze the influence of a backpack load of 4 kg on the gait and postural sway of elementary school children.

**Material and methods:**

In this prospective study, a group of 12 elementary school children aged between 7 and 10 years without neurological or orthopedic problems participated. The measurements included a clinical examination, three-dimensional gait analysis with electromyographic recordings and measurement of postural sway on a force plate.

**Results:**

The backpack load, on average 15% of the body weight, led to a slower walking speed, shorter step length and increased double-support phase. Increased anterior pelvic and trunk tilt, as well as hip flexion were also observed. Furthermore, the muscle activity and postural sway of the children were affected by the increased load.

**Conclusions:**

School backpacks weighing 4 kg caused changes in gait, muscle activity, posture and stability in elementary school children. Due to the weight of the backpack, the centre of mass shifted backwards and the children became less stable. During gait, this was compensated by increased anterior pelvic and trunk tilt and increased hip flexion. The activity of the paraspinal muscles was decreased and indicates that the backpack is carried passively. This may cause a negative long-term effect.

## Hintergrund

In den vergangenen Jahren nahmen Rückenschmerzen und Fehlhaltungen bei Kindern und Jugendlichen zu [4]. Als Ursachen werden u. a. eine geringe körperliche Aktivität und das tägliche Tragen eines Schulrucksacks angenommen [15]. Die generelle Empfehlung für das Maximalgewicht eines Schulrucksacks liegt bei 10–15 % des Körpergewichts [17], was bei Messungen in der Mehrheit der Studien deutlich überschritten wurde. Cavallo et al. stellten fest, dass mehr als ein Viertel der Schülerinnen einer vierten Klasse einen Rucksack mit mehr als 15 % des Körpergewichts trugen [5], während andere Studien sogar von einer durchschnittlichen Rucksackbelastung von über 20 % des eigenen Körpergewichts ausgingen [17, 29].

Neben Rückenschmerzen können auch eine gestörte Schulterhaltung, Fehlkontrolle des Gleichgewichts sowie Wirbelsäulendeformitäten mit der Belastung durch den Schulrucksack bei Jugendlichen einhergehen, was sich wiederum auf das Gangbild auswirken könnte [9, 16, 24, 25].

Obwohl das Thema des Tragens eines Schulrucksacks seit langem von wissenschaftlichem Interesse ist und die Traglast biomechanische Anpassungen in Rücken und Körperhaltung bewirkt [17], konnte eine Kausalität für das Auftreten von Rückenschmerzen bisher nicht eindeutig belegt werden [32, 34].

Ziel der vorliegenden prospektiven Studie war es, den Einfluss einer Rucksacklast auf das Gangbild, die Muskelaktivität und die Standstabilität bei gesunden Grundschulkindern zu untersuchen.

## Methode

Nach Genehmigung durch die institutionelle Ethikkommission nahmen zwölf Kinder zwischen 7,0 und 10,1 Jahren ohne neurologische oder orthopädische Probleme an der Studie teil. Das Kollektiv wird in Tab. 1 dargestellt.

**Table 1:** 

Proband	Alter (Jahre)	Geschlecht	Körpergröße (cm)	Körpergewicht (kg)	Rucksackgewicht (% vom Körpergewicht)
1	8,2	M	139,0	31,8	12,6
2	8,2	W	128,5	26,2	15,3
3	8,3	W	134,5	27,2	14,7
4	7,8	W	122,5	20,5	19,5
5	9,2	W	143,0	28,9	13,8
6	8,3	W	129,0	24,6	16,3
7	7,9	M	123,5	22,5	17,8
8	10,1	W	145,5	44,5	9,0
9	8,7	M	134,0	27,7	14,4
10	9,6	M	134,0	30,1	13,3
11	7,3	M	124,0	23,7	16,9
12	7,0	W	124,5	24,0	16,7
Mittelwert	8,4 (±0,9)	M = 5; W = 7	131,8 (±7,8)	27,6 (±6,2)	15,0 (±2,7)

Die Messungen umfassten eine klinische Untersuchung, eine dreidimensionale (3D) Ganganalyse mit oberflächenelektromyografischen (EMG) Aufzeichnungen und die Prüfung des ruhigen Stehens auf einer Kraftmessplatte.

### Klinische Untersuchung

Die klinische Untersuchung umfasste die Messung der unteren Extremitäten und der Wirbelsäule. Die anthropometrischen Daten beinhalteten Gewicht, Größe, Beinlänge (Distanz Spina iliaca anterior superior zum Malleolus medialis), Distanz Spina iliaca anterior superior zum Trochanter major, Kniebreite (lateraler und medialer femoraler Epikondylus), Malleolenbreite, Tibiatorsion (Rotation der Tibia) und femorale Antetorsion (Schenkelhalsachse im Vergleich zur hinteren Kondylenebene des distalen Femurs). Die Messung der Augenhöhe wurde zur Stabilitätsmessung benötigt. Zusätzlich wurde anamnestisch nach sportlicher Betätigung, Unfällen und Operationen, Rücken‑/Schulterschmerzen und Krankheiten gefragt.

### Ganganalyse

**Figure 1:**
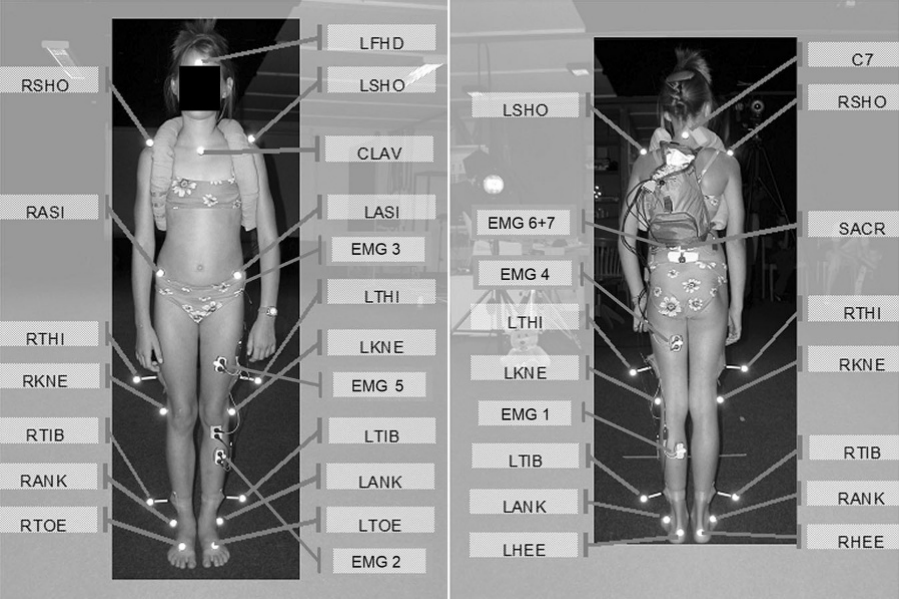

Die 3D-Ganganalyse erfolgte unter Verwendung des optoelektronischen Bewegungsanalysesystems VICON 460 (Vicon, Oxford, UK), das mit sechs hochauflösenden Infrarotkameras ausgestattet ist, die in einem Kreis um den Gehweg angeordnet sind (Aufzeichnungsfrequenz 120 Hz). Die 3D-Bewegungsspuren von passiven reflektierenden Markern (14 mm) wurden unter Verwendung der VICON-Workstation-Software (Vicon, Oxford, UK) rekonstruiert. Das Plug-in-Gait-Markierungsmodell [18] wurde verwendet, um 3D-Gelenkwinkel der unteren Extremitäten zu berechnen (Abb. 1). Zusätzlich wurden fünf Marker an (1) linker Schulter-Akromion, (2) rechter Schulter-Akromion, (3) Jugulargrube, (4) Dornfortsatz Halswirbelkörper C7 und (5) Stirnmitte angebracht. Aus den zusätzlichen Markern 1 bis 4 wurde ein fiktiver Mittelpunkt berechnet. Ebenso wurde ein fiktiver Mittelpunkt aus den drei Beckenmarkern (Sakrum, linke und rechte Spina iliaca anterior superior) berechnet. Die Linie dieser beiden fiktiven Punkte repräsentiert die Oberkörpervorneigung in Grad relativ zur Horizontalen.

Die Kinder wurden gebeten, barfuß, geradeausschauend und mit einer selbst gewählten Geschwindigkeit zu gehen, ohne den Kopf zu drehen. Insgesamt wurden jeweils sechs Gehversuche einer 10 m geraden Strecke ohne und sechs Gehversuche mit 4 kg Sand im Rucksack aufgezeichnet. Bei dem Rucksack handelte es sich um ein sehr kleines Standardmodell und nicht um einen klassischen Schulrucksack, damit die Marker adäquat sichtbar waren. Die Daten der 3D-Gelenksrotationen, wie auch die EMG-Daten, wurden zeitnormalisiert zu dem Gangzyklus. Der Gangzyklus ist die Zeitspanne zwischen zwei Fußauftritten desselben Fußes, wobei der Zeitpunkt des Zehenabhebens den Gangzyklus in die Stand- und Schwungphase unterteilt.

### Elektromyographie (EMG)

Die EMG wurde an den folgenden Muskeln des dominanten Beins (ermittelt durch einbeiniges Hüpfen) gemessen: 1) M. gastrocnemius medialis, 2) M. tibialis anterior, 3) M. gluteus medius, 4) M. semitendinosus, 5) M. rectus femoris und 6 + 7) rechte und linke Paraspinalmuskulatur in Höhe von L2. Eine Erdungselektrode wurde auf der Tuberositas tibiae angebracht. Dazu wurde die Haut an den Elektrodenstellen rasiert und mit Alkohol entfettet. Die EMG-Elektroden (10 mm Durchmesser, 22 mm Elektrodenabstand) wurden auf die Haut in Faserrichtung des Zielmuskels entsprechend des SENIAM-Standards geklebt [14].

Die Signale wurden bei einer Abtastrate von 2520 Hz digital (10–700 Hz Bandbreite) mit dem Zebris/Bioversion-Oberflächen-EMG-System (Zebris Medizintechnik GmbH, Tübingen, Deutschland) gefiltert. Das EMG-System war mit dem VICON-System gekoppelt und die Daten wurden zeitsynchron mit den Bewegungsdaten aufgenommen. Die EMG-Rohdaten wurden gefiltert (Bandpassfilter 20–500 Hz), Vollwellen-gleichgerichtet und geglättet (4th Order Butterworth Filter). Für jedes Kind wurde die Amplitude der EMG-Daten der beiden Versuche mit 0 und 4 kg Rucksackgewicht auf das Signalmaximum der 0‑kg-Mittelwert-Kurve normalisiert. Sämtliche EMG-Daten wurden mit der Software MATLAB analysiert (The MathWorks Inc., Natick, MA, USA).

### Stabilitätsmessung

Die Messung der Standstabilität erfolgte in der Mitte einer Kraftmessplatte (Typ 9287A, Kistler Instrumente AG, Winterthur, Schweiz). Die Kinder wurden gebeten, so still wie möglich zu stehen und auf einen 3 m entfernten Fixierungspunkt in Augenhöhe zu schauen. Für beide Versuchsbedingungen (0 und 4 kg) wurden drei Versuche von 10 s bei unveränderter Fußposition aufgenommen (Abtastrate: 600 Hz). Der Mittelwert der Position des Kraftmittelpunkts in anterior-posteriorer Richtung relativ zu der Fußlängsachse (Linie zwischen Zehen- und Fersenmarker) über die 10 s wurde berechnet. Ebenso wurde die Standardabweichung dieser Position des Kraftmittelpunkts in anterior-posteriorer und mediolateraler Richtung berechnet. Sie ist ein Maß für die Stabilität des freien Stehens [10].

### Statistische Analyse

Für jeden Proband und Parameter wurde jeweils der Mittelwert aus sechs Wiederholungen, respektive drei Wiederholungen bei der Stabilitätsmessung, berechnet. Für die kinematische und EMG-Analyse wurden die Daten des dominanten Beins verwendet. Eine Normalverteilung konnte für die Zeit-Raum-Parameter, die durchschnittliche Oberkörpervorneigung, die durchschnittliche Beckenkippung und die Daten der Stabilitätsmessung mittels des Shapiro-Wilk-Tests bestätigt werden. Daraufhin wurden diese Parameter mit einem zweiseitigen, gepaarten (Rucksack ohne und mit 4 kg Gewicht) Student-t-Test (*p* < 0,05) statistisch geprüft und anschließend die statistische Power berechnet. Die Daten werden als Gruppenmittelwerte und Standardabweichung (SD) dargestellt. Für den statistischen Vergleich der kinematischen und EMG-Kurven wurde das Statistical Parametric Mapping (SPM) angewendet (zweiseitiger gepaarter t‑Test, *p* < 0,05). Alle statistischen Analysen wurden mit der Software MATLAB und der Open-Source Software spm1D 0.4 (www.spm1d.org) durchgeführt. Mittels der SPM-Methode können Signifikanz-Cluster in den ganzen Kurvenverläufe definiert werden. Hierbei wird unter Verwendung der Random-Field-Theorie der *p*-Wert an Mehrfachvergleiche angepasst.

## Ergebnisse

An der vorliegenden Studie nahmen zwölf gesunde Kinder (sieben Mädchen, fünf Jungen) im Durchschnittsalter von 8,4 Jahren (7,0–10,1 Jahren) ohne neurologische oder orthopädische Auffälligkeiten teil (Tab. 1). Die durchschnittliche Körperlänge betrug 132 cm (SD 8 cm) und das Durchschnittsgewicht 27,6 kg (SD 6,2 kg). Vor der Teilnahme gaben alle Kinder und ihre Eltern ihre schriftliche Einverständniserklärung gemäß der Helsinki-Erklärung ab, die von der institutionellen Ethikkommission genehmigt wurde.

### Zeit-Raum-Parameter

Verglichen mit dem leeren Rucksack wiesen die Probanden mit der 4‑kg-Traglast eine reduzierte Ganggeschwindigkeit (0 kg: 1,16 [SD 0,17] m/s; 4 kg: 1,11 [SD 0,14] m/s; *p* = 0,014, Power: 0,99) und eine verkürzte Schrittlänge (0 kg: 0,54 [SD 0,07] m; 4 kg: 0,51 [SD 0,05] m; *p* = 0,008, Power: 0,99) bei unveränderte Kadenz (0 kg: 126 [SD 9] Schritte/min; 4 kg: 129 [SD 8] Schritte/min; *p* = 0,405) auf. Außerdem bewirkte die Rucksacklast eine erhöhte Doppelunterstützungsphase (0 kg: 18,7 [SD 2,9] s; 4 kg: 20,9 [SD 2,2] s; *p* = 0,003, Power: 0,75), d. h. die Phase im Gangzyklus mit beiden Füßen den Boden berührend, auf Kosten der Einzelunterstützungsphase (0 kg: 41,3 [SD 1,2] s; 4 kg: 39,7 [SD 1,3] s;* p* < 0,001, Power: 0,40). Mit dem Rucksackgewicht von 4 kg erfolgte der Zeitpunkt des Zehenabhebens verspätet (0 kg: 59,5 [SD 1,3] % Gangzyklus; 4 kg: 60,5 [SD 1,3] % Gangzyklus; *p* = 0,010, Power: 0,60) und die Standphase war verlängert.

### Kinematische Daten

**Figure 2:**
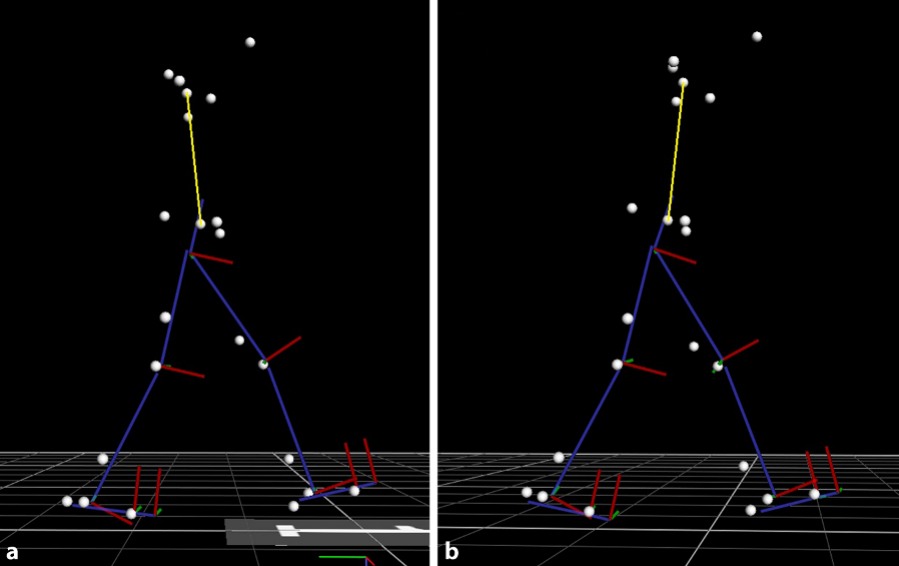

**Figure 3:**
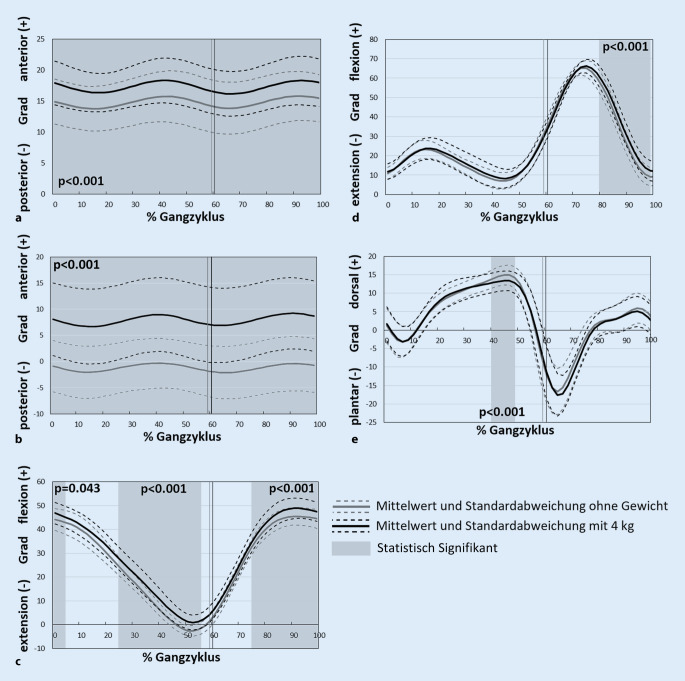

Mit einer Rucksacklast von 4 kg war der Oberkörper durchschnittlich 9,1° (SD 3,0) (*p* < 0,001, Power: 0,99) mehr nach vorne geneigt im Vergleich zu ohne Last (Abb. 3b). Die Abb. 2 zeigt exemplarisch für einen Proband das Markermodell mit der Vorneigung in der Sagittalebene mit und ohne Gewicht.

Das Becken kippte durch die Rucksacklast um durchschnittlich 2,5° (SD 1,9) (*p* < 0,001, Power: 0,98) nach vorne im Vergleich zu ohne Last (Abb. 3a). Über die Mehrheit des Gangzyklus war eine erhöhte Hüftflexion durch die Traglast festzustellen (Abb. 3c). Dies zeigte sich am Anfang (0–4 %) und Ende (74–100 %) des Gangzyklus sowie in der Mitte der Standphase (24–56 % Gangzyklus). Die Bewegungsmuster der Kniegelenke waren unverändert in der Standphase, nach dem Befüllen des Rucksacks aber mehr flektiert in der Schwungphase zwischen 80 und 98 % des Gangzyklus (Abb. 3d). Im oberen Sprunggelenkbereich zeigte sich bei beiden Versuchsbedingungen mit und ohne Traglast ein Fersen-Ballen-Gang, wobei in der terminalen Standphase zwischen 40 und 48 % des Gangzyklus mit der Traglast eine verminderte Dorsalflexion beobachtet werden konnte (Abb. 3e).

### EMG

**Figure 4:**
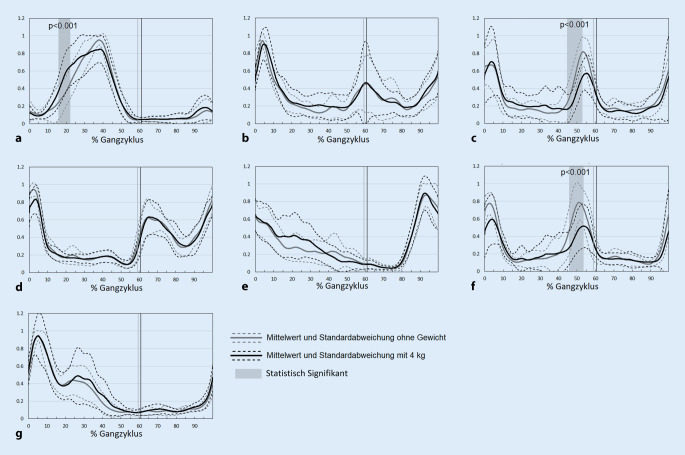

Die De- und Repolarisationsvorgänge der Muskeln zeigten durch die Traglast ein erhöhtes Aktivitätsmuster des M. gastrocnemius medialis in der mittleren Standphase (16–22 % des Gangzyklus) (Abb. 4a). Die Muskelaktivität der ipsilateralen und kontralateralen Paraspinalmuskulatur wurde durch das Rucksackgewicht in der zweiten Hälfte der terminalen Standphase (45–53 % des Gangzyklus) verringert (Abb. 4c, f). Die Aktivitäten der übrigen Muskeln zeigten keine signifikanten Veränderungen (Abb. 4b, d, e, g).

### Stabilität

**Figure 5:**
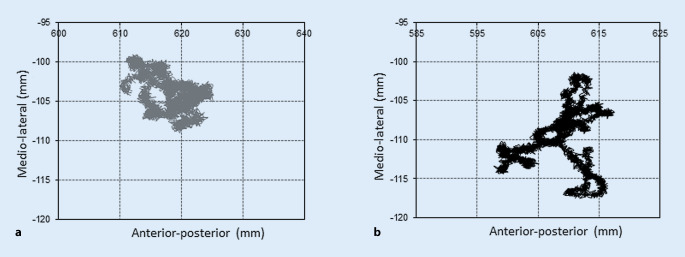

Die Abb. 5 zeigt exemplarisch die grafische Darstellung des Verlaufs des Kraftangriffspunktes während des ruhigen Stehens einer 7‑jährigen Probandin ohne Gewicht (Abb. 5a) und mit 4 kg Gewicht im Rucksack (Abb. 5b). Durch die Traglast erhöhte sich in der Probandengruppe sowohl in anterior-posteriorer als auch mediolateraler Richtung die durchschnittliche Schwankung des Kraftangriffspunktes signifikant. Die Standardabweichung der Position über 10 s erhöhte sich von durchschnittlich 6,3 (SD 2,2) mm ohne Last auf 7,5 (SD 2,7) mm mit Last (*p* = 0,019, Power: 0,91) in anterior-posteriorer Richtung, respektive von 3,8 (SD 1,8) mm ohne Gewicht auf 5,0 (SD 1,9) mm mit Gewicht (*p* < 0,001, Power: 0,99) in mediolateraler Richtung. Die durchschnittliche anterior-posteriore Position des Kraftangriffspunktes lag ohne Rucksackgewicht 2,6 cm (SD 3,0) anterior zur Hälfte der Fußlängsachse und war mit 4 kg Rucksackgewicht unverändert 2,8 cm (SD 3,3; *p* = 0,47, Power: 0,99).

## Diskussion

Bis vor einigen Jahrzehnten waren Rückenschmerzen im Kindes- und Jugendalter eine Seltenheit. In der ersten Folgebefragung (KIGGS Welle 1) berichteten Krause et al. jedoch von einer Prävalenz wiederholter Rückenschmerzen in den letzten 3 Monaten von 17,7 % bei 11- bis 17-Jährigen, von 3,8 % in der Altersgruppe 7–10 Jahre und von 1 % in der Altersgruppe 3–6 Jahre [22]. Somit spielt mit steigendem Alter eine Rückenschmerzproblematik eine zunehmende Rolle bis hin zu 80–90 % in der erwachsenen Bevölkerung, was einen gravierenden sozioökonomischen Faktor ausmacht.

Es wird angenommen, dass der Grundstein für die Rückengesundheit in der Kindheit gelegt wird. Die gesellschaftliche Sorge um den gegenwärtigen und zukünftigen gesunden Rücken von Kindern ist weit verbreitet [25]. Einen möglichen Einflussfaktor stellt das tägliche Tragen eines Schulrucksacks und die damit wiederholte (unphysiologische) Belastung der Wirbelsäule dar. Die Korrelation von Rucksacklast und Rückenschmerzen oder falscher Körperhaltung wird diskutiert, jedoch fehlen in den meisten Ländern spezifische Vorschriften zum Gewicht und zur Tragezeit des Schulrucksackes [15, 17].

In unserer Studie über den Effekt des Rucksackgewichts auf die Muskelaktivität und Gelenkrotation beim Gehen sowie die Standstabilität von Grundschulkindern, lag die Traglast bei durchschnittlich 15,0 (SD 2,7) % des Körpergewichts. Dieser Wert ist vergleichbar mit [3, 11] bzw. niedriger [29] als in vorherigen Studien zur Belastung durch den Schulrucksack angegeben.

Die Rucksacklast führte in unserer Studie durch eine verkürzte Schrittlänge zu einer reduzierten Ganggeschwindigkeit. Eine mögliche Erklärung für das verlangsamte Gehen ist die Auswirkung des Rucksackgewicht auf die Gangstabilität [28, 31]. Konsequenterweise konnten wir auch auf der Kraftmessplatte eine erhöhte Standinstabilität beobachten, sobald der 4 kg schwere Rucksack getragen wurde. Auch die Verlängerung der Doppelunterstützungsphase, bei der beide Füße gleichzeitig am Boden verbleiben, ist ein Indiz für die Zunahme der Instabilität. Eine reduzierte Ganggeschwindigkeit mit verkürzter Schrittlänge und verlängerter Doppelunterstützungsphase konnten auch in anderen Studien sowohl bei Kindern als auch Erwachsenen gezeigt werden [1, 6, 27, 33].

Kinematisch wiesen die Kinder in unserer Studie durch die 4‑kg-Traglast eine erhöhte Beckenvorwärtskippung auf, welche eine stärkere Hüftflexion bei gleichbleibendem absolutem Bewegungsumfang in der Hüfte zur Folge hatte. Das Gewicht des Rucksacks verlagerte den Körperschwerpunkt nach hinten und bewirkte eine Anpassung des Oberkörpers und des Beckens nach vorne, um das Gleichgewicht zu halten [8]. Auch andere Studien beobachteten bei Schulkindern mit einer Rucksacklast zwischen 8,5 und 20 % des Körpergewichts eine Vorneigung des Oberkörpers mit einer erhöhten vorderen Beckenkippung und ausgeprägter Hüftflexion [16, 21, 24]. Auch bei älteren Jugendlichen und Erwachsenen konnte eine traglastbedingte Rumpfvorneigung beobachtet werden [6, 12, 30]. Dabei intensivierte sich die Rumpfvorneigung mit der Rucksacklast [16, 24]. Häufig wird die Rucksacklast durch eine Hohlkreuzhaltung kompensiert [20]. Die Beckenkippung nach vorne induziert eine Hyperlordose der Lendenwirbelsäulen, die bei anhaltendem Zustand Lumbalschmerzen auslösen kann. Als alternativer Kompensationsmechanismus gilt der Rundrücken, wobei das Becken aufgerichtet und die Lendenlordose aufgehoben wird und bei vorgeschobenem Kopf ein nach vorne gewölbter thorakaler Rundrücken entsteht. Für beide Mechanismen, die Hohlkreuzhaltung als auch der thorakale Rundrücken, sollte eine Stärkung der Rückenmuskulatur als Präventionsmaßnahme gegen Rückenschmerzen diskutiert werden [19]. Auch das Design des Schulrucksacks in Bezug auf Ergonomie und Passgenauigkeit an der Rückenpartie sowie die Tragegewohnheiten, insbesondere die symmetrische Verteilung der Last auf beide Schultern, spielen eine Rolle [2, 26].

Die Rucksacklast führte auch zu einigen Änderungen in der Muskelaktivität. Die Paraspinalmuskulatur zeigte eine ersten Aktivitätsspitze beim initialen Bodenkontakt und eine zweite Spitze beim initialen Bodenkontakt des kontralateralen Fußes. Dies korrespondiert mit den beiden Doppelunterstützungsphasen am Anfang und gegen Ende der Standphase. Die Aktivität der ipsi- und kontralateralen Paraspinalmuskeln wurde durch die erhöhte Traglast beim Aufsetzen des kontralateralen Fußes in der zweiten Doppelunterstützungsphase vermindert. In dieser Gangzyklusphase wird das Körpergewicht auf das Gegenbein verlagert, wobei sowohl das Becken als auch der Oberkörper sich leicht nach hinten neigen. Möglicherweise wird die Gewichtsverlagerung auf das Gegenbein und die Becken-Rumpf-Kontrolle unter anderem durch die Paraspinalmuskeln kontrolliert. Weil der Körperschwerpunkt durch die Last auf dem Rücken nach hinten verschoben wird [7], nimmt die Aktivität der Rückenmuskulatur ab und die Aktivität der Bauchmuskeln zu, um den Körperschwerpunkt entgegen der Last halten zu können. Die Paraspinalmuskulatur muss dabei die Wirbelsäule weniger stabilisieren [23], was darauf hindeutet, dass der schwere Rucksack eher passiv und nicht aktiv durch Muskelarbeit getragen wird. Devroey et al. zeigten gleiche Resultate mit einer verminderten Aktivität der Rückenmuskulatur und einer Zunahme der Aktivität der Bauchmuskulatur, was auf eine verminderte Ko-Kontraktion zwischen Bauch- und Rückenmuskulatur hindeutet, mit eventuell negativen Langzeitfolgen [8]. In unserer Studie ließ sich diese verminderte Paraspinalmuskelaktivität nur bei der Gewichtsverlagerung auf den kontralateralen Fuß in der zweiten Doppelunterstützungsphase, nicht aber bei der Gewichtsverlagerung vom kontralateralen Fuß auf den ipsilateralen Fuß zu Beginn des Gangzyklus aufweisen. Harman et al. zeigten mit zunehmender Ganggeschwindigkeit eine erhöhte Paraspinalmuskelaktivität beim Tragen eines Rucksacks [13]. Da unsere Ergebnisse eine reduzierte Ganggeschwindigkeit durch das Tragen der Rucksacklast demonstrierten, kann auch die Ganggeschwindigkeit die reduzierte Muskelaktivität beeinflusst haben.

Zusammenfassend lässt sich feststellen, dass ein Rucksackgewicht von 15 % des Körpergewichts bei Grundschulkindern zu Änderungen von Gang, Muskelaktivität, Haltung und Standstabilität führte. Dies war eine Momentaufnahme unter Laborbedingungen. Über dauerhafte Schädigungen oder Haltungsänderungen im Langzeitverlauf kann keine Aussage getroffen werden. Weitere Limitationen der Studie sind die relativ geringe Anzahl an Probanden (*n* = 12), die nicht erfolgte Messung der Muskelaktivität der Bauchmuskeln und die fehlende Beachtung weiterer Einflussfaktoren, wie zum Beispiel die individuelle Körperkonstitution und Fitness der Probanden. Zudem wurde für alle Probanden eine Traglast von 4 kg und nicht eine an das Probandengewicht angepasste Traglast gewählt. Mit diesem Vorgehen sollte eine möglichst reelle Situation abgebildet werden.

Da in der vorliegenden Arbeit eindeutig Veränderungen auf das muskuloskelettale System festgestellt werden konnten, ist eine Reduktion des Schulrucksackgewichtes z. B. durch einen doppelten Schulbüchersatz, Online-Bücher oder Schulspinde, sowie eine tägliche Reduktion unnötigen Materials aus dem Schulrucksack wünschenswert. Zu diskutieren sind weiterhin alternative Schulrucksacktransportmittel, wie beispielsweise der Fahrradkorb.

## Abkürzungen

EMG: Elektromyographie
KIGGS: Studie zur Gesundheit von Kindern und Jugendlichen in Deutschland
SD: Standardabweichung
SENIAM: Surface ElectroMyoGraphy for the Non-Invasive Assessment of Muscles
SPM: Statistical Parametric Mapping
